# A structural equation model to test a conceptual framework of oral health in Japanese edentulous patients with an item weighting method using factor score weights: a cross-sectional study

**DOI:** 10.1186/s12903-018-0527-1

**Published:** 2018-04-27

**Authors:** Eijiro Yamaga, Yusuke Sato, Shunsuke Minakuchi

**Affiliations:** Gerodontology and Oral Rehabilitation, Department of Gerontology and Gerodontology, Graduate School of Medical and Dental Sciences, Tokyo Medical and Dental University, Yushima Bunkyo-ku, Tokyo, 113-8549 Japan

**Keywords:** Oral health quality of life, Edentulous, Conceptual model, Structural equation model, Factor score weight, Generalization

## Abstract

**Background:**

To investigate Locker’s multidimensional model of oral health in Japanese edentulous patients with an item weighting method using factor score weights, which is more accurate than the sum scoring method. A previous study tested Locker’s model in edentulous elders in the UK, using empirical evidence from the Short-Form Oral Health Impact Profile (OHIP-14). Investigating the model using the OHIP for edentulous subjects (OHIP-EDENT), which contains 19 items suitable for these patients, may complement that study. Testing Locker’s model in Japanese patients may support generalization of the model.

**Methods:**

A total of 394 patients who were edentulous in both arches and visited the Dental Hospital of Tokyo Medical and Dental University for new complete dentures were recruited. This cross-sectional study had a non-probabilistic sampling design and included the following: data collection; application of the new item weighting method that involves hierarchical confirmatory factor analysis (CFA) to derive factor score weights for each item, using the bootstrap method, to check the significance of the factor score weights; and empirical testing of Locker’s conceptual model of oral health in Japanese edentulous patients, using structural equation modelling analysis with the bootstrap method for precise estimations and model generation.

**Results:**

Factor score weights derived from CFA were significant. After item weighting, the initial model was analyzed and found to have an inconsistent direct path (functional limitation to disability). This path was eliminated from the model and the modified model was re-run. All effects were significant. The model showed acceptable fit on indices including the model chi-squared, standardized root-mean-square residual, root mean-square error of approximation, goodness-of-fit index, comparative fit index, and *P*-value.

**Conclusions:**

Our findings showed an empirical fit to Locker’s model in Japanese edentulous patients when using the item weighting method, which was more accurate than the sum scoring method. These results could contribute to the generalization of Locker’s model.

**Trial registration:**

The experimental procedures were published in the University hospital Medical Information Network (UMIN) Center (UMINCTR Clinical Trial, Unique trial Number: UMIN000028711).

## Background

Oral health-related quality of life (OHRQoL) is a multidimensional construct. OHRQoL has been researched mostly based on Locker’s conceptual model of oral health [1]. Locker proposed a scientific model that aims to specify the complicated consequences of oral disease on quality of life. Nevertheless, no study, except for that by Baker [2], has investigated Locker’s model explicitly using empirical evidence. In that study [2], data for three samples (general adults, edentulous elders, and patients with xerostomia) were analyzed and the short version of the Oral Health Impact Profile (OHIP-14) [3] was used as the measure.

The OHIP [4] is often used to evaluate the multidimensional construct of OHRQoL. However, the large number of items included makes it difficult for participants to complete the survey. Therefore, the OHIP-14 was designed and has been widely adopted to assess the association between OHRQoL and a clinical intervention [5]. However, because of a floor effect, the OHIP-14 cannot determine improvements in edentulous persons following clinical intervention [6]. The OHIP-EDENT is a shortened version of the OHIP, which includes 19 items suitable for edentulous persons. By including an item on chewing and eating difficulty, the OHIP-EDENT could detect OHRQoL changes in edentulous persons with new or different prostheses [6]. In the present study, the Japanese version of the Oral Health Impact Profile for edentulous subjects (OHIP-EDENT-J), a cross-culturally adapted scale, was used [7].

Historically, the numbers of edentulous persons in developed countries have been decreasing. However, given the present ageing of societies, the need for treatment of edentulous persons is not anticipated to decrease overall [8]. The World Health Organization recommends that socioepidemiological research focusing on high-risk groups, including edentulous patients, is needed in order to improve the health of older adults [9]. Further, Critchlow and Ellis [10] concluded that the evidence base in complete denture research suffers from an insufficient number of well-conducted studies. Using the OHIP-14, Baker [2] succeeded in indicating that Locker’s conceptual model of oral health is supported by empirical evidence in edentulous elders as well as in the general adult population. An investigation applying the OHIP-EDENT to Locker’s model in edentulous patients may complement Baker’s study.

Item weighting is a process by which the relative weight of events can be expressed. Using a weighted scoring system, the discriminant validity of OHIP was improved to a small extent [11]; however, it does not have good cost-performance [12]. That is, item weighting is a time-consuming process that offers only slight improvement of discriminant validity. On the other hands, DiStefano et al. [13] reported that sum scoring was a non-refined method because its score does not necessarily indicate adequate contribution to the factor (e.g., negative factor loading). Zucoloto et al. [14] also regarded sum scoring as an inaccurate method, and proposed a second-order or third-order model for derivation of the scores on the subscales and an overall score for the measure that adequately improves the accuracy of estimation of the construct using the structural equation modelling (SEM) method. SEM is a powerful multi-variable analytical method that can present direct and indirect effects separately and express complicated relationships in a path diagram [15].

The aim of this study was to investigate Locker’s conceptual model of oral health in Japanese edentulous patients with the OHIP-EDENT-J using SEM with the item weighting method proposed by Zucoloto et al. in order to generalize Locker’s model. The following hypotheses were tested: functional limitations would be related to disability, which would be related to handicap, which in turn would be related to pain and discomfort; both pain and discomfort would be associated with disability; and pain would be related to discomfort. These hypotheses were adopted as the conceptual model of oral health in a sample of edentulous elders in a previous study by Baker [2].

## Methods

The study was conducted in three stages: 1) collection of data; 2) deriving weighting formulae from hierarchical confirmatory factor analysis (CFA) to improve the accuracy of the estimation [14]; and 3) empirical testing of Locker’s conceptual model of oral health in Japanese edentulous patients with the OHIP-EDENT-J [7] using SEM analysis after item weighting derived from CFA. A cross-sectional design with non-probabilistic sampling was adopted.

### Participants

The participants were systemically healthy persons who were edentulous in both arches and visited the Dental Hospital of Tokyo Medical and Dental University requesting new complete dentures during the period from January 2009 to April 2015. The exclusion criteria included no existing denture or dentures and non-attendance before measurements. Three hundred and ninety-four patients were recruited for the study. One patient was hospitalized, another one was withdrawn, 49 had missing data, leaving 343 patients (87.1%, mean age 76.3 ± 8.3 years) for analysis. The patient characteristics, oral condition, and quality of previous dentures were investigated by calibrated prosthodontists with more than 4 years of clinical experience, during the creation of the new complete dentures (Table 1). The method devised by Cawood and Howell [16] was employed to assess the residual ridge forms. Denture stability and retention were estimated using the Kapur method [17]. Jaw relation was estimated by investigating whether premature contact was existing or not in centric relation. The assessments of patient characteristics, oral condition, and quality of previous dentures are part of the screening process for patients requesting new complete dentures, and thus were not purely for purpose of this study. All subjects provided written informed consent to participate in this study.

**Table 1:** Patient characteristics, oral condition, and quality of previous dentures

Variable	Participants (*N* = 343), *n* (%)	
Sex		
Male	140 (40.8)	
Female	203 (59.2)	
Edentulous period (years)		
< 1	77 (22.4)	
1 to < 3	28 (8.2)	
3 to < 5	18 (5.3)	
5 to < 10	42 (12.2)	
10+	174 (50.7)	
Forgotten	4 (1.2)	
Age of present denture (years)		
< 5	127 (37.0)	
5 to < 10	132 (38.5)	
10+	81 (23.6)	
Forgotten	3 (0.9)	
Ridge form (Cawood & Howell classification)^a^		
	Maxilla	Mandible
Class II	6 (1.7)	18 (5.3)
Class III	249 (72.6)	103 (30.0)
Class IV	57 (16.6)	81(23.6)
Class V	28 (8.2)	106 (30.9)
Class VI	1 (0.3)	31 (9.0)
Others	2 (0.6)	4 (1.2)
Denture stability (Kapur method)^b^		
0	37 (10.8)	132 (38.5)
1	109 (31.7)	139 (40.5)
2	197 (57.4)	72 (21.0)
Denture retention (Kapur method)^c^		
0	24 (7.0)	134 (39.1)
1	62 (18.1)	105 (30.6)
2	85 (24.8)	57 (16.6)
3	172 (50.1)	47 (13.7)
Jaw relation		
Premature contact (−)	232 (67.6)	
Premature contact (+)	111 (32.4)	

^a^Class II, immediately post extraction; Class III, well-rounded ridge form, adequate in height and width; Class IV, knife-edge ridge form, adequate in height and inadequate in width; Class V, flat ridge form, inadequate in height and width; Class VI, depressed ridge form, with some basal loss evident. ^b^Scoring system: 0, no stability, when a denture base demonstrates extreme rocking on its supporting structures under pressure; 1, some stability, when a denture base demonstrates moderate rocking on its supporting structures under pressure; 2, sufficient stability, when a denture base demonstrates slight or no rocking on its supporting structures under pressure. ^c^Scoring system: 0, no retention, when a denture is seated in place, it displaces itself; 1, minimum retention, when a denture offers slight resistance to vertical pull and little or no resistance to lateral force; 2, moderate retention, when a denture offers moderate resistance to vertical pull and little or no resistance to lateral force; 3, good retention, when a denture offers maximum resistance to vertical pull and sufficient resistance to lateral force

### OHIP-EDENT-J

To investigate the multidimensional construct of OHRQoL, the OHIP was assessed using the OHIP-EDENT-J [7]. The OHIP-EDENT-J has 19 items and consists of seven subscales (functional limitation, pain, psychological discomfort, physical disability, psychological disability, social disability, and handicap) and is based on Locker’s model [1]. Functional limitation is defined as the extent of depression of function of body parts or systems. The definition of discomfort is the self-assessment of physical and psychological distress, including pain and other feelings that are not directly observable. Disability is expressed as three dimensions of well-being (physical, psychological, and social). Handicap is concerned with the social effects of disease, which are broader than those of disability [1]. Participants were asked how many times they had experienced the impact of each item in the previous month using a scale ranging from 0 (never) to 4 (very often).

### Factor score weights

To improve the accuracy of estimation of the construct, we employed hierarchical CFA using SEM analysis [14, 15]. The SEM analysis was conducted with AMOS (SPSS Statistics version 17.0, SPSS Inc., Chicago, IL). Given that many authors have indicated their calculation of the OHIP by summing all items, the existence of the third-order factor (OHIP) is presumably assured [14]. Therefore, we performed CFA using a third-order hierarchical CFA model and derived a formula whereby the third-order factor (OHIP) could be estimated. The third-order model has been described in the literature [14]. The scores derived from the formula can obtain a more accurate estimation than the simple summing method. In detail, the weighting formula derived from the third-order model included factor score weights for items 1–19. The product of the factor score weight and average deviation of item score for the raw data was adopted as the final item score to investigate the hypothesized model. Evaluation of the significance of factor score weights was conducted using bias-corrected bootstrapped 95% confidence intervals (CIs) [18] based on 1000 replications. The method used to assess the model fit of CFA is described in the following paragraph.

### Testing the Locker model

Locker’s conceptual model of oral health in edentulous patients was empirically investigated using SEM. The hypothesized model was that used in a previous study of edentulous patients by Baker [2]. The maximum likelihood method is adopted for estimation of free parameters and requires data that have a normal distribution. More than 1.0 of absolute value of kurtosis was regarded as non-normal distribution. The bootstrap method can also be used to determine parameter estimates in data that have a non-normal distribution [18]. Parameter estimates of the direct and indirect effects were determined using the bootstrapping method with 1000 iterations.

### Estimation of model fit

We assessed model fit to the data using five indices commonly used in SEM analysis, i.e., the chi-squared test and *P*-value, the standardized root-mean-square residual (SRMR), the root mean-square error of approximation (RMSEA), the comparative fit index (CFI), and the goodness-of-fit index (GFI) [15]. As the chi-squared value increases and the *P*-value consequently decreases, the fit of the model becomes increasingly worse. A ‘larger’ *P*-value indicates a ‘better’ model fit. SRMR values less than 0.08 are generally considered to be favorable [19, 20]. In general, an RMSEA less than 0.05 indicates a close fit, values between 0.05 and 0.08 indicate a reasonable fit, and an RMSEA more than 0.1 indicates a poor fit [21]. A GFI and a CFI of 1.0 indicates a complete model fit. Generally, a GFI and a CFI greater than 0.95 indicates a good fit [19, 20].

### Strategy in model specification

There are some strategies involved in specification and evaluation of the model. MacCallum and Austin [21] proposed three SEM analysis strategies: (a) a strictly confirmatory strategy, in which a single a priori model is investigated; (b) a model generation strategy, in which an initial model is fitted to the data and then modified as necessary until the fit is adequate; and (c) an alternative model strategy, in which various a priori models are studied. We employed (a) a strictly confirmatory strategy for CFA and (b) a model generation strategy for the Baker model.

## Results

The means, medians, and standard deviations (SDs) of the observed variables before weighting and Pearson’s correlations between observed variables after weighting are shown in Table 2. There were no correlations with high coefficients (> 0.85), indicating that multicollinearity did not occur in the SEM analysis.

**Table 2:** Pearson’s correlations after item weighting

	Pearson’s correlations				Summary measures			
	Functional limitation	Physical pain	Psychological discomfort	Disability	Mean	Median	SD	Score range
Functional limitation	–	–	–	–	5.93	6	3.04	0–12
Physical pain	0.812***	–	–	–	6.54	6	3.91	0–16
Psychological discomfort	0.694***	0.812***	–	–	3.21	3	2.26	0–32
Disability	0.545***	0.676***	0.736***	–	7.47	7	5.97	0–8
Handicap	0.438***	0.544***	0.589***	0.756***	1.72	2	1.86	0–8

*SD* standard deviation, the means, SDs, and ranges indicate the scores for each variable before item weighting. ***P < 0.001

Univariate kurtosis in items 2, 7, 10, 13, and 15–19 (CFA section), handicap (Baker model section after weighting), and multivariate kurtosis (CFA and Baker model section) indicated a non-normal distribution.

### Factor score weights

**Fig. 1:**
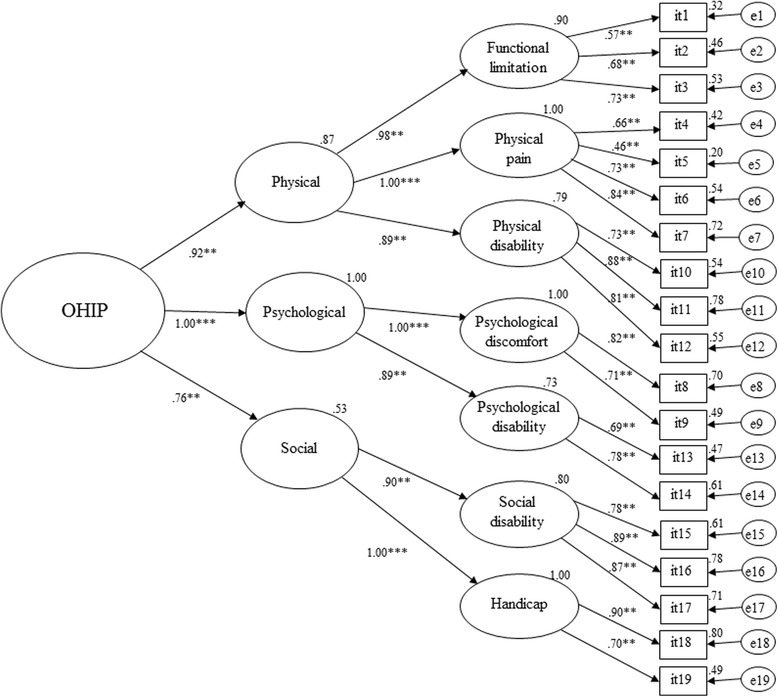
The confirmatory factor analysis model to derive factor score weights. Bootstrap standardized direct effects for third-order hierarchical model of the Oral Health Impact Profile for edentulous subjects (OHIP-EDENT). Numbers on the upper right-hand side of the rectangles and ellipses represent the coefficient of determination associated with each structural equation. **P < 0.01, ***P < 0.001

We derived the weighting formula from hierarchical CFA in which the third-order model was employed using raw data (OHIP item score). The CFA model and the bootstrap standardized estimates of direct effect are shown in Fig. 1. The fit indices were as follows: chi-squared = 897.03 (146 degrees of freedom), *P* < 0.001, CFI = 0.83, GFI = 0.76, RMSEA = 0.12 (90% CI 0.11–0.13), and SRMR = 0.089. The fit of the model was poor. The bootstrap standardized estimates and the standard error and CI values for the factor score weights of each item (OHIP) are shown in Table 3. All factor score weights were significant. Based on the model, the item scores for the third-order factor (OHIP) can be estimated by the following formula [14]:

$$ {\displaystyle \begin{array}{l}\mathrm{OHIP},\mathrm{y}=0.025\mathrm{it}1+0.035\mathrm{it}2+0.045\mathrm{it}3+0.037\mathrm{it}4+0.019\mathrm{it}5+0.049\mathrm{it}6+0.092\mathrm{it}7+\\ {}0.202\mathrm{it}8+0.114\mathrm{it}9+0.019\mathrm{it}10+0.050\mathrm{it}11+0.032\mathrm{it}12+0.101\mathrm{it}13+0.115\mathrm{it}14+\\ {}0.015\mathrm{it}15+0.037\mathrm{it}16+0.029\mathrm{it}17+0.100\mathrm{it}18+0.022\mathrm{it}19\end{array}} $$

**Table 3:** Factor score weights for each item derived from the CFA model using the bootstrap method

Item	Β	Bootstrap SE	Bias-corrected 95% CI
1	0.025**	0.008	0.012 / 0.044
2	0.035**	0.012	0.015 / 0.065
3	0.045**	0.014	0.022 / 0.079
4	0.037**	0.010	0.019 / 0.059
5	0.019***	0.006	0.009 / 0.035
6	0.049**	0.016	0.022 / 0.083
7	0.092**	0.034	0.039 / 0.166
8	0.202**	0.044	0.112 / 0.280
9	0.114**	0.033	0.054 / 0.180
10	0.019**	0.008	0.007 / 0.040
11	0.050**	0.016	0.022 / 0.086
12	0.032**	0.011	0.014 / 0.056
13	0.101**	0.056	0.041 / 0.261
14	0.115**	0.055	0.051 / 0.251
15	0.015**	0.008	0.005 / 0.040
16	0.037**	0.019	0.015 / 0.089
17	0.029**	0.016	0.010 / 0.074
18	0.100*	0.032	0.053 / 0.177
19	0.022*	0.013	0.007 / 0.060

*OHIP* Oral Health Impact Profile, *CFA* confirmatory factor analysis, *SE* standard error, *CI* confidence interval, **P < 0.01, ***P < 0.001

### Testing the locker model

**Fig. 2:**
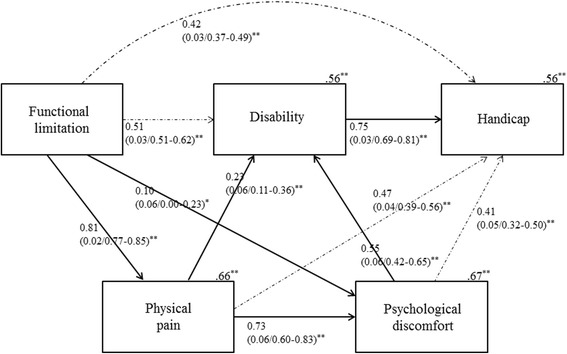
The (modified) final Baker model. Bootstrap standardized estimates (SE/BC 95% confidence intervals) for the modified Baker model (*n* = 343) after item weighting using factor score weights. Numbers on the upper right-hand side of the rectangles represent the coefficient of determination. Residual errors are eliminated for concise visual interpretation. Solid (full) lines indicate direct paths and dashed (dotted) lines indicate indirect paths. **P < 0.01

The main (Baker) model for the a priori hypotheses showed an acceptable fit on all indices: the GFI was 1.00, the CFI was 1.00, the RMSEA was 0.00 (90% CI 0.00–0.08), the SRMR was 0.013, the chi-squared value (3 degrees of freedom) was 2.139, and the *P*-value was 0.544 with weighted data. However, the direct effect of functional limitation on disability was a minus quantity, which was inadequate considering the consistency of association (worse functional limitation was associated with improving disability). Therefore, the path was deleted from the initial hypothesized (modified Baker) model. When the modified Baker model was re-run, the data supported Locker’s conceptual model [1] in terms of the estimation of effects and fit indices. The fit indices of the modified Baker model were as follows: GFI = 1.00, CFI = 1.00, RMSEA = 0.00 (90% CI 0.00-0.08), SRMR = 0.013, chi-squared value (4 degrees of freedom) = 3.431, and *P*-value = 0.488. Therefore, all five criteria were met. The modified Baker model accounted for 66% of the variance in pain, 66% in discomfort, 56% in disability, and 57% in handicap. The bootstrap standardized estimates, standard error values, and bias-corrected 95% CIs of direct effects and indirect effects are shown in Fig. 2.

## Discussion

The present findings support Locker’s conceptual model of oral health [1] and complement a previous well-designed study [2]. Both the study by Baker and the present study show that Locker’s model can be generalized to various samples, including both edentulous patients and the general adult population, and that in both UK and Japanese edentulous sample, Locker’s model can be applied.

By empirical analysis of the structure of a model, a theoretical model may be evaluated as highly sophisticated when compared with models that explain the nature of directional relationships between elements [22]. SEM is a powerful analytical method that is useful for investigating complex relationships like the structure of the elements of OHIP and presents the percentage of variance of the variables. In this study, the final (modified Baker) model explained 66% of the variance in pain, 66% in discomfort, 56% in disability, and 57% in handicap. That is, 34%–44% of the variance was not expressed in the model. Baker [2] referred to coping strategies, social support, sense of coherence, and negative affectivity as key contextual factors that may have improved interpretability. Moreover, we propose that elements of personality, such as neuroticism and life satisfaction, play an important role in oral health. Fenlon et al. [23] demonstrated that neuroticism had an influence on satisfaction with complete dentures and Yamaga et al. [24] indicated that satisfaction with complete dentures was associated with OHIP. Therefore, neuroticism may influence oral health. Locker et al. [25] showed a significant relationship between life satisfaction and oral health in older adults. Therefore, life satisfaction may be related to oral health, especially in edentulous patients. If these variables had been included in this study model, more variation in OHIP elements may have been obtained.

In the present study, the final (modified Baker) model indicated higher fit indices than those indicated in the previous study [2] in edentulous patients. The *P*-value in the previous study was 0.350 and in the present study was 0.488. This may be because we used the OHIP-EDENT, which succeeded in eliminating the ceiling effect by including items relevant to chewing and eating difficulty [12], and not the sum scoring method but the item weighting method using hierarchical CFA with SEM analysis.

Jenkinson [26] indicated that the item weighting method is not so useful, whereas Zucoloto et al. [14] affirmed the correctness of item weighting. Jenkinson showed that measurements of health status are not significantly improved by weighting of items [26]. On the other hand, Zucoloto et al. [14] referred to the usefulness of the scoring method that adopted CFA with SEM. The theoretical concepts of physical, psychological, and social as second-order, or OHIP as third-order, have been discussed in the literature [27]. However, to date, its construct validity could not be tested by CFA analysis, which is important for accurate estimation. Therefore, further study is needed. The sum scoring method does not necessarily express the degree of effect of the score on the factor (OHIP). On the other hand, this weighting method can reflect how the score contributed to the factor (OHIP).

SEM analysis requires a large sample size (individuals) to obtain a precise estimation in free parameters. No absolute criteria for sample size exist in the literature. However, the complexity of the model is thought to be critical for sample size (individuals). A larger sample (individuals) was needed because the model was more complex and included more free parameters. In general, 20 individuals per free parameter is considered the desirable sample size [15]. Given that the hypothesized (Baker) model in the present study had 12 free parameters to be estimated, 240 individuals was considered the minimum adequate sample size. The third-order hierarchical (CFA) model had 44 free parameters to be estimated. Therefore, 880 individuals were needed. On the other hand, sample size (individuals) more than 200 was recommended in the field of social psychology for SEM analysis in the point of absolute criteria based on the general guide [15]. Both models met this recommendation.

In this study, the third-order model was used to interpret the multidimensional construct of OHRQoL and adjust item scores. It is possible to use various models, including CFA, to derive weight factor scores and understand the construct. For example, Baker [28] constructed a model for use in housebound edentulous elders in which functional (OHIP) was used as the latent variable (first order), physical, psychological, and social as indicator variables, and the covariance between the residual error of the psychological and social items was added. In the literature, the relevance of general health perception, functional (OHIP), and symptom status was investigated using a two-stage approach to SEM analysis [29]. Therefore, a more macroscopic view might be required to capture the multidimensional construct of OHRQoL rather than detailed elements, such as physical pain, as employed in this study. While a number of possible models exist, the third-order model was used to derive factor score weights because the third-order model covers all possible models and is not perfect but has been adequately tested in the literature [14]. A model fit was poor in the CFA model from which factor score weights were derived. However, bias-corrected bootstrapped 95% confidence intervals showed significance; the sample size recommendation in terms of absolute criteria was met. Moreover, the bootstrapping method had been recommended as the best approach for small-moderate sample sizes [18].

In the final (modified Baker) model, the direct effect of functional limitation on disability was not examined because of apparent inconsistency in the amount of direct effect. That is, it appears that more functional limitation decreases disability as derived from the initial hypothesized model, whereas functional limitation has a significant large indirect effect on disability. To wit, in edentulous patients, functional limitation influences disability indirectly rather than directly. This is because of the strong direct link between functional limitation and pain (0.81) and the indirect link between pain and discomfort (0.73). Clinically, it may be that functional limitation (e.g., dentures not fitting) has an indirect influence on disability (e.g., avoidance of eating) via pain or discomfort rather than a direct influence. In terms of general statistical principles, not all the potential direct relationships were incorporated (the parsimony principle) [15].

The main limitation of this study is its cross-sectional rather than longitudinal design. Thereby, a causal relationship could not be shown. Further studies including intervention would be required to determine the relationship between change in scores for before and after outcome variables. According to the theory of response shift [30], a follow-up response may be influenced by new information not available at the time of the initial response. On outcome evaluation, the response shift causes bias that confuses the meaning of the score. To eliminate this source of bias, future studies should include a longitudinal design.

## Conclusions

The results of the present study show an empirical fit to Locker’s model in Japanese edentulous patients by an item weighting method using factor score weights, which has more accuracy than the sum scoring method. This finding may contribute to the generalization of Locker’s model.

## Abbreviations

CFA: Confirmatory factor analysis
CFI: Comparative fit index
CI: Confidence interval
GFI: Goodness-of-fit index
OHIP-14: The Short-Form Oral Health Impact Profile,
OHIP-EDENT: OHIP for edentulous subjects
OHIP-EDENT-J: The Japanese version of the Oral Health Impact Profile for edentulous subjects
OHRQoL: Oral health-related quality of life
RMSEA: Root mean-square error of approximation
SD: Standard deviation
SEM: Structural equation modeling
SRMR: Standardized root-mean-square residual
UMIN: The University hospital Medical Information Network
